# Flash Communication: Properties and Applications of a Pentavalent Bromoantimony Lewis Acid

**DOI:** 10.1021/acs.organomet.5c00468

**Published:** 2026-01-15

**Authors:** Alexander C. Mehnert, Chenggang Jiang, Brendan L. Murphy, You Jiang, François P. Gabbaï

**Affiliations:** Department of Chemistry, 14736 Texas A&M University, College Station, Texas 77843-3255, United States

## Abstract

While normally reluctant toward oxidation, SbBr_3_ can be oxidized with 3,4,5,6-tetrachloro-1,2-benzoquinone (*o*-chloranil) in the presence of a Lewis base like the bromide anion or triphenylphosphine oxide (PPh_3_O) to form the tetrachlorocatecholate (cat^Cl^) derivatives [SbBr_4_(cat^Cl^)]^−^ ([**2**-Br]^−^) and SbBr_3_(cat^Cl^)•OPPh_3_ (**2**•OPPh_3_), respectively. Structural, spectroscopic, and computational data indicate that the SbBr_3_(cat^Cl^) fragment is similarly, if slightly less, Lewis acidic than the previously reported SbCl_3_(cat^Cl^) fragment. The SbBr_3_/*o*-chloranil system, as well as its previously reported counterpart SbCl_3_/*o*-chloranil, were tested as Lewis acid catalysts for C–O bond metathesis and polycarbonate depolymerization. These results identified SbX_3_/*o*-chloranil pairs (X = Cl, Br) as simple main-group platforms for C–O bond cleavage chemistry.

## Graphical abstract

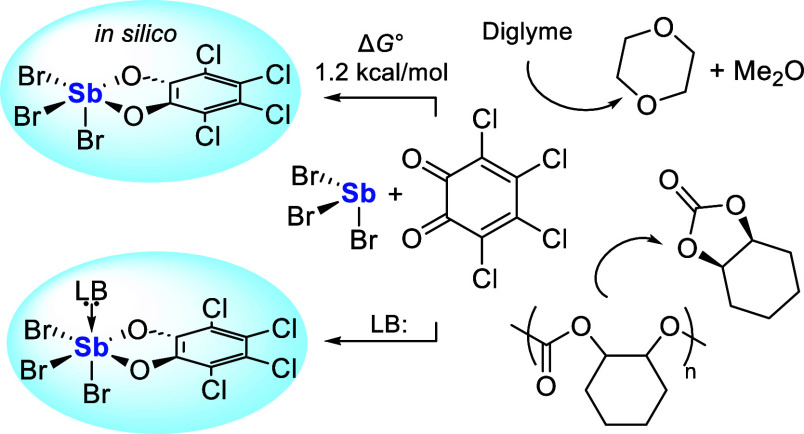

Among group 15 elements, or pnictogens, Sb­(V) compounds stand out as particularly potent Lewis acids^1–3^ with remarkable affinity for anions in solution.^4,5^ This property is derived from the strong attractive interactions that an Sb­(V) center forms with an incoming Lewis base while minimizing Pauli repulsions due to long, flexible primary bonds.^6^ Early explorations of such compounds can be found in the works of Olah and Gillespie who used antimony pentahalides to generate superacids^7–9^ and for the activation of C–F bonds.^10,11^ However, these compounds, analogs of which have been recently used for C–H bond activation chemistry,^12,13^ are corrosive and react quite vigorously with water, relegating them primarily to reactions in inert and rigorously dry conditions. Fortunately, organoantimony­(V) compounds often retain respectable Lewis acidity when arylated,^14–16^ tempering their reactivity toward ambient conditions which sometimes affords these compounds water tolerance.^17^ One class of organoantimony­(V) compounds that our group has taken interest in is the catecholatostiborane, which can be easily generated *via* the reaction of a stibine precursor and an *ortho*-quinone such as *o*-chloranil to produce compounds of the general formula Ar_3_Sb­(catecholate) (Scheme 1a).^18–21^ Indeed, catecholatostiboranes have been used in a number of applications including small molecule activation, catalysis and anion transport.^22–28^

**Scheme 1:**
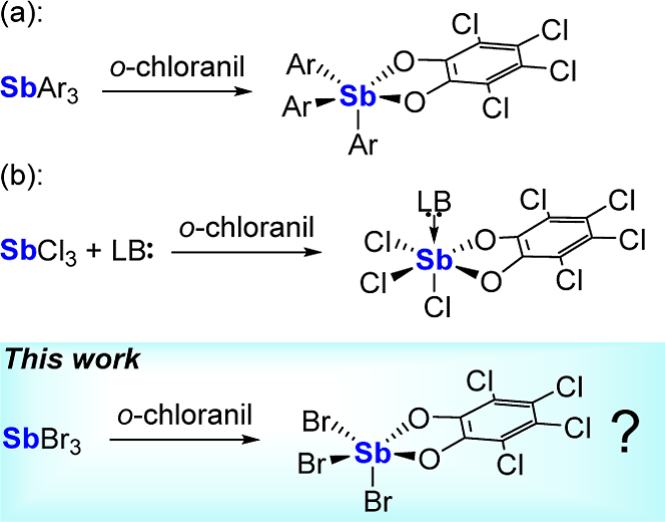
Oxidation of Trivalent Stibines to Pentavalent Stiboranes by *o*-chloranil and Objective of the Present Study

Building on these precedents, we recently reported the oxidation of SbCl_3_ by *o*-chloranil in the presence of a Lewis base, which traps the otherwise elusive SbCl_3_(cat^Cl^) (cat^Cl^ = tetracholorcatecholate) fragment (**1**) (Scheme 1b).^29^ This strategy provides a potent, easy-to-handle surrogate of the highly Lewis acidic yet corrosive SbCl_5_ that can mediate transformations like the polymerization of tetrahydrofuran and the activation of aliphatic C–F bonds. In the course of those studies, we were surprised to find a dearth of information regarding the fundamental properties of high-valent bromoantimony compounds. For example, SbCl_5_ can be easily produced *via* the oxidation of SbCl_3_ with Cl_2_, thus its fundamental chemistry^30–33^ has been robustly explored. SbBr_5_, on the other hand, does not form when SbBr_3_ and Br_2_ are combined,^34,35^ even though the [SbBr_4_]^+^ cation^36^ and [SbBr_6_]^−^ anion^37,38^ are known. Herein, we detail a strategy to easily access a pentavalent bromoantimony scaffold and we assess its Lewis acidity experimentally and computationally. Moreover, we also explore the potential of SbX_3_(cat^Cl^) (X = Cl, Br) fragments as Lewis acidic platforms for C–O bond cleavage chemistry.

To begin, we carried out quantum-chemical calculations on the target Lewis acid SbBr_3_(cat^Cl^) (**2**). Geometry optimization was performed using the PBEh-3c/def2-mSVP level of theory^39^ within Orca 6.0.1 ^40^ (see Supporting Information for full details), revealing a pseudo-square pyramidal coordination environment around the central antimony atom. A thermodynamic analysis in the gas phase indicates that the association of SbBr_3_ with *o*-chloranil is slightly endergonic (Δ*G* = +1.2 kcal mol^–1^), suggesting the disfavored formation of **2** under standard conditions (Figure 1). Indeed, attempts to observe the *in situ* formation of **2** in CDCl_3_ *via* ^13^C­{^1^H} NMR spectroscopy only detected free *o*-chloranil (Figure S1), mirroring the reactivity previously reported for **1**.

**Figure 1:**
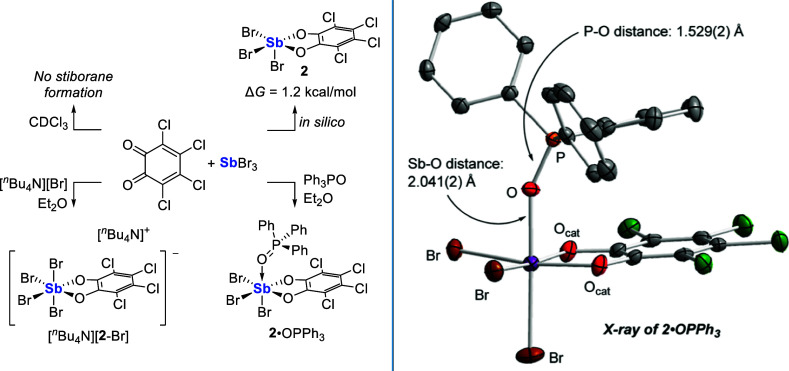
Left: reactivity of the SbBr_3_/*o*-chloranil system reported in this work. Right: solid-state structure of **2**•OPPh_3_. Hydrogen atoms are omitted for clarity. Thermal ellipsoids are drawn at 50% probability level.

However, we contended that we may be able to trap the fragment with a Lewis base in a manner similar to the SbCl_3_/*o*-chloranil pair (Figure 1). In a test of this idea, treatment of equimolar amounts of SbBr_3_, *o*-chloranil, and [^*n*^Bu_4_N]­[Br] in diethyl ether (Et_2_O) produced a grayish powder. Evidence of oxidation could be seen *via* ^13^C­{^1^H} NMR spectroscopy by following the upfield shift of the C–O resonance from 169.0 ppm for free *o*-chloranil in CDCl_3_ to 144.8 ppm for the corresponding catechol in CD_2_Cl_2_. Recrystallization yielded dusky, yellow crystals which allowed us to confirm, using single-crystal X-ray diffractometry (scXRD), the formation of [^*n*^Bu_4_N]­[SbBr_4_(cat^Cl^)] ([^*n*^Bu_4_N]­[**2**-Br], Figure S4). The structure of [**2**-Br]^−^ is very similar to that found for [**1**-Cl]^−^, as can be seen by the average Sb–O_cat_ distances of both anions being nearly identical (2.010(3) Å for [**1**-Cl]^−^; 2.020(4) Å for [**2**-Br]^−^: 2.020(4) Å).^29^ Also notable is that the average Sb–Br bond distances found in [**2**-Br]^−^ (2.5266(8) Å) are comparable to those found for SbBr_6_^–^ (∼2.55 Å).^37^ We were unfortunately unable to isolate sufficient quantities of pure [^*n*^Bu_4_N]­[**2**-Br] for full characterization, though this reaction serves as proof of principle.

To compare the Lewis acidity of **2** to other pentavalent antimony systems, SbBr_3_, *o*-chloranil, and triphenylphosphine oxide (PPh_3_O) were then mixed in Et_2_O. Following recrystallization under ambient conditions, the adduct **2**•OPPh_3_ was isolated as dark red crystals and fully characterized by NMR spectroscopy, scXRD, and elemental analysis. The ^31^P­{^1^H} NMR spectrum of this adduct in CDCl_3_ finds a single resonance at 44.5 ppm, markedly downfield from those of free OPPh_3_ (29.1 ppm) and SbPh_3_(cat^Cl^)•OPPh_3_ (30.6 ppm).^24,29^ Notably, this resonance is in the same range as Cl_5_Sb•OPPh_3_ (47 ppm)^41^ and **1**•OPPh_3_ (46.0 ppm). Further insight came from the solid-state structure of **2**•OPPh_3_ which, like **1**•OPPh_3_, crystallizes as the *fac*-isomer with the Ph_3_PO positioned *trans* to a halide ligand (Figure 1). The resulting Sb-O_P_ distance of 2.041(2) Å is near the sum of the covalent radii of the two elements (Σ*r*_cov_(Sb,O): 2.03 Å),^42^ indicating a strong linkage that is of a similar length to that found in the corresponding structure of **1**•OPPh_3_ (avg. Sb–O distance: 2.0276(14) Å).^29^ The P–O bond of the bound Ph_3_PO Lewis base (P–O: 1.529(2) Å) is lengthened compared to free Ph_3_PO (P–O: 1.484(1) Å),^43^ denoting a substantial polarization of the motif. In fact, this bond nears that found for **1**•OPPh_3_ (avg. P–O distance: 1.5348(15) Å)^29^ and approaches the P–O bond distance in [MeOPPh_3_]^+^ (P–O: 1.562(1) Å).^44^ In total, these measurements suggest that **2** is potently Lewis acidic and is similarly, if slightly less, Lewis acidic than its chloride analog **1**.

Prompted by these results, we sought comparison of Lewis acidity strength between **1** and **2** *in silico*. To simplify the calculation, dimethyl ether (Me_2_O) was employed as the base and the free energies of the reaction in eq 1 were computed. This calculation returned a value of −9.4 kcal mol^–1^ for **1** and −7.3 kcal mol^–1^ for **2** at 298 K, suggesting that the Lewis acidity of **2** is slightly weaker than that of **1**. This differential was also supported by the computed Sb–O_ether_ bond distances of 2.34 Å for **1**•OMe_2_ and 2.41 Å for **2**•OMe_2_ in the optimized structures of the adducts. However, we should bear in mind that **1** and **2** are not accessible at room temperature. For this reason, we also computed the isodesmic reaction shown in eq 2 which accounts for the fact that **1** and **2** are endergonic species. The Δ*G* value of −0.5 kcal mol^–1^computed for this reactionindicates a negligible difference in the Lewis acidity of the SbCl_3_/*o*-chloranil and SbBr_3_/*o*-chloranil systems.
1

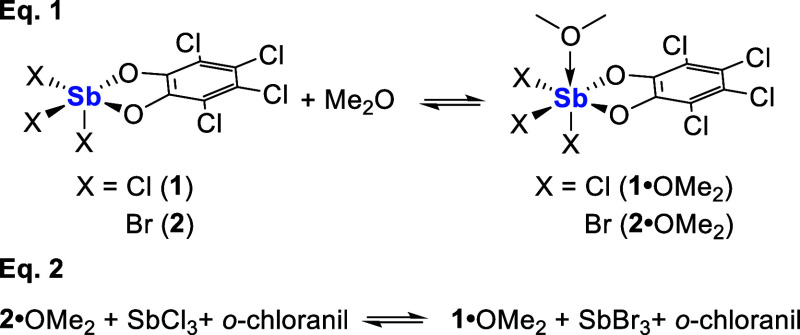




Motivated by these findings, we began to study the potential of **2** as a Lewis acid catalyst (Figure 2). We first turned our attention to diglyme as a simple model for C–O bond metathesis, a substrate that has previously been used with silicon Lewis acids.^45–47^ Treatment of diglyme with 5 mol% of SbBr_3_/*o*-chloranil in *o*-dichlorobenzene at 115 °C led to the formation of 1,4-dioxane in 40% yield after 19 h with Me_2_O as a stoichiometric byproduct (Figure 2, top). This result parallels the recent work of Greb, who demonstrated the *in situ* generation of a silicon-based polyether depolymerization catalyst *via* SiCl_4_ and perhalogenated catechols.^48^ Interestingly, the SbCl_3_/*o*-chloranil system under identical conditions showed higher conversion (63%), suggesting that the bromide-based system is less efficient in facilitating this C–O bond metathesis. Although less efficient than the chloride system, the SbBr_3_/*o*-chloranil pair still behaves as a strong Lewis acid capable of cleaving robust C–O bonds.

**Figure 2:**
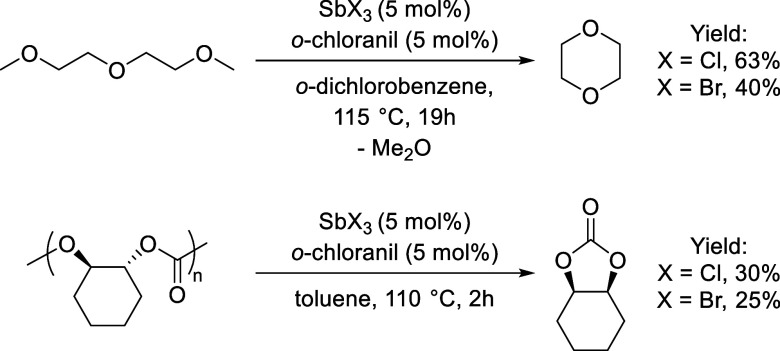
Top: catalytic and stoichiometric reactivity promoted by SbX_3_/*o*-chloranil (X = Cl, Br) including ring-closing C–O bond metathesis of diglyme. Bottom: depolymerization of poly­(cyclohexene carbonate) promoted by SbX_3_/*o*-chloranil systems (X = Cl, Br).

Encouraged by these results, we then decided to test whether these systems would also promote depolymerization of CO_2_-based polycarbonates. Previous work with triarylborane catalysts has shown that efficient depolymerization of such a polymer requires a strong Lewis acid; tris­(pentafluorophenyl)­borane efficiently catalyzes depolymerization to cyclic carbonates whereas the less Lewis acidic triphenylborane is inactive under comparable conditions.^49,50^ In view of these precedents, we anticipated that both SbX_3_/*o*-chloranil systems could be competent in promoting the degradation of poly­(cyclohexene carbonate) (PCHC), a widely studied CO_2_-based polymer.^51^ Treatment of PCHC with 5 mol % SbBr_3_/*o*-chloranil in toluene at 110 °C for 2 h afforded cyclohexene carbonate in 25% yield, while the analogous SbCl_3_/*o*-chloranil system gave 30% yield under the same conditions (Figure 2, bottom). MALDI-TOF analysis of the polymer residue after the reaction showed that the number-average molar mass collapsed from over 30,000 to around 2000, accompanied by a broad distribution of short oligomers (Figures S10 and S11). The pronounced reduction in chain length at modest overall conversion implies a random chain-scission process rather than a chain-end unzipping mechanism.^52^ Extending the reaction time to 4 h under the same conditions increased the yield to 49% for **1** and 31% for **2** (Figures S14 and S15). However, no substantial increase in yield was observed upon prolonged heating beyond 4 h, possibly due to catalyst degradation (Figures S16 and S17). In all cases, *cis*-cyclohexene carbonate was obtained selectively as the product, consistent with the stereoselectivity reported for the borane systems. Although the SbBr_3_/*o*-chloranil system is slightly less efficient than its chloride analogue, it remains sufficiently potent to promote the depolymerization of PCHC to *cis*-cyclohexene carbonate.

In summary, we demonstrate that SbBr_3_ can be oxidized with *o*-chloranil in the presence of a Lewis base to generate the rare pentavalent bromoantimony fragment **2**. Through scXRD and NMR spectroscopy, we find that **2** is similarly Lewis acidic to its trichloride counterpart **1**, a finding that is buttressed by calculations. Finally, we show that both SbCl_3_/*o*-chloranil and SbBr_3_/*o*-chloranil promote C–O bond cleavage reactions, including the depolymerization of PCHC to cyclic carbonate. Applications of other strongly Lewis acidic antimony­(V) compounds toward other depolymerization schemes are under exploration in our laboratory and will be reported in due course.

## Supplementary Material





## Notes

Additional experimental and computational details (PDF).
Optimized structures in (XYZ)
