# Supplementary material for: Flash Communication: Properties and Applications of a Pentavalent Bromoantimony Lewis Acid
Source: Organometallics. 2026 Jan 15;45(3):334–8. doi: 10.1021/acs.organomet.5c00468 (PMC12892314; doi:10.1021/acs.organomet.5c00468)
Supplement: Supplementary file 1 [file om5c00468_si_001.pdf]

Flash Communication: Properties and Applications of a Pentavalent Bromoantimony Lewis Acid

Alexander C. Mehnert, Chenggang Jiang, Brendan L. Murphy, You Jiang, François P. Gabbaï*

Department of Chemistry, Texas A&M University, College Station, Texas 77843-3255, United States
*Corresponding author, email: francois@tamu.edu

Supporting Information

This PDF file includes

Contents:

1. Synthesis and characterization
- **1.1. General considerations**
- **1.2. Synthetic procedures**
- **1.3. NMR spectra and solid-state structures**

2. Computational methods
- **2.1. Geometry optimizations**
- **2.2. *In silico* reaction thermochemistry**
- **2.3. Energy summary**

3. Catalytic screening
- **3.1. General procedure for the C-O bond metathesis of diglyme**
- **3.2. General procedure for the depolymerization of PCHC**
- **3.3. Characterization of the products**

1. Synthesis and characterization

1.1. General considerations

All experiments were carried out in an ambient environment unless otherwise noted. Chemicals were purchased from commercial suppliers and used without further purification. Et_2O and toluene were dried over Na/K and distilled prior to use. Diglyme and 1,2-dichlorobenzene were dried by refluxing over CaH_2. All other solvents were ACS reagent grade and used as received. Poly(cyclohexene carbonate) was prepared according to literature procedures,[1] and the average molecular weight was determined to be 30600 by gel permeation chromatography (GPC). NMR spectra were recorded at room temperature using a Bruker Avance 500 spectrometer (500 MHz for ^{1}H and 126 MHz for ^{13}C),), a Bruker Avance 500 NMR spectrometer (126 MHz for ^{13}C), or a Bruker Ascend 400 NMR spectrometer (400 MHz for ^{1}H and 162 MHz for ^{31}P). Chemical shifts are given in ppm and are referenced against residual solvent signals (^{1}H, ^{13}C) or external standards. Elemental analyses (EA) were performed at Atlantic Microlab (Norcross, GA).

All crystallographic measurements were performed at 110(1) K using a Bruker D8 QUEST diffractometer (Mo-Kα radiation, $\lambda = 0.71073$ Å). In each case, a specimen of suitable size and quality was selected and mounted onto a nylon loop. Integrated intensity information for each reflection was obtained by reducing the data frames using APEX3.[2] The semi-empirical methods SADABS[3] was used for the absorption corrections. The structures were solved by direct methods using ShelXT[4] and refined against F^2 with anisotropic temperature-dependent parameters for all non-hydrogen atoms using ShelXL[5] using the Olex2 interface.[6] All H-atoms were geometrically placed and refined using a riding model. Diamond4 was used for final data presentation. CCDC 2504518-2504519 contains the supplementary crystallographic data for this paper. These data can be obtained free of charge *via* www.ccdc.cam.ac.uk/data_request/cif, or by emailing data_request@ccdc.cam.ac.uk, or by contacting The Cambridge Crystallographic Data Centre, 12 Union Road, Cambridge CB2 1EZ, UK; fax: +44 1223 336033.

1.2. Synthetic procedures

Attempted synthesis of **2**: *o*-chloranil (200 mg, 0.81 mmol) was dissolved in 0.7 mL benchtop $CDCl_3$ and a ^{13}C NMR was taken. $SbBr_3$ (294 mg, 0.81 mmol) dissolved in 1.0 mL benchtop $CDCl_3$ was added to the *o*-chloranil solution. The normally deep red color of *o*-chloranil deepened to a purple color and a ^{13}C NMR was taken. The resonances corresponding to free *o*-chloranil were the only ones detected by this technique.

Synthesis of [$^{n}Bu_4N$][**2**-Br]: $SbBr_3$ (201 mg, 0.56 mmol), *o*-chloranil (137 mg, 0.56 mmol), and [$^{n}Bu_4N$][Br] (179 mg, 0.56 mmol) were suspended in Et_2O (20 mL) and stirred overnight, resulting in the precipitation of a greyish-green powder. This powder was collected on a frit, washed with petroleum ether (2 × 15 mL), and dried *in vacuo*, with a crude yield of 361 mg. Single crystals of [$^{n}Bu_4N$][**2**-Br] suitable for X-ray diffractometry were grown as dusky, yellow blocks *via* layering Et_2O onto a CH_2Cl_2 solution of the compound. This reaction was reproduced several times, yet it did not yield a sufficient amount of pure material for full characterization. We have included the spectra of the crude material as Figures S2-3.

^{1}H NMR (500 MHz, CD_2Cl_2) δ 3.14-3.10 (m, 8H, $^{n}Bu_4N$ CH_2), 1.65-1.59 (m, 8H, $^{n}Bu_4N$ CH_2), 1.43-1.40 (m, 8H, $^{n}Bu_4N$ CH_2), 1.01 (t, J = 7.3 Hz, 12H, (m, 8H, $^{n}Bu_4N$ CH_3).

^{13}C{^{1}H} NMR (126 MHz, CD_2Cl_2) δ 144.8, 121.7, 116.5, 59.7, 24.5, 20.3, 14.0.

Synthesis of **2**•$OPPh_3$: Under a nitrogen atmosphere, $SbBr_3$ (40 mg, 0.11 mmol) and *o*-chloranil (27 mg, 0.11 mmol) were dissolved in anhydrous Et_2O (40 mL) and stirred for 1 h, giving a red-colored solution. Triphenylphosphine oxide (31 mg, 0.11 mmol) was dissolved in Et_2O (20 mL) before being added to the reaction mixture. The resulting solution was then stirred at room temperature for another 4 h. The solvent was removed *in vacuo*, affording a dark red crude solid. The final product was purified by recrystallization from CH_2Cl_2/pentane. Yield: 26 mg (27%, 2.9 x 10^{-2} mmol).

^{1}H NMR (500 MHz, $CDCl_3$) δ 7.68–7.56 (m, 9H, OPPh_3) 7.53–7.49 (m, 6H, OPPh_3).

^{13}C{^{1}H} NMR (126 MHz, $CDCl_3$) δ 143.4, 134.5 (d, J= 2.7 Hz), 133.3 (d, J= 11.8 Hz), 129.3 (d, J= 13.3 Hz), 125.0, 124.2, 122.2, 116.7.

^{31}P{^{1}H} NMR (162 MHz, $CDCl_3$) δ 44.6 (s).

Elemental analysis calculated for $C_{24}H_{15}Br_3Cl_4O_3PSb$: C 32.55, H 1.71; found: C 32.44, H 1.58.

1.3. NMR spectra and solid-state structures

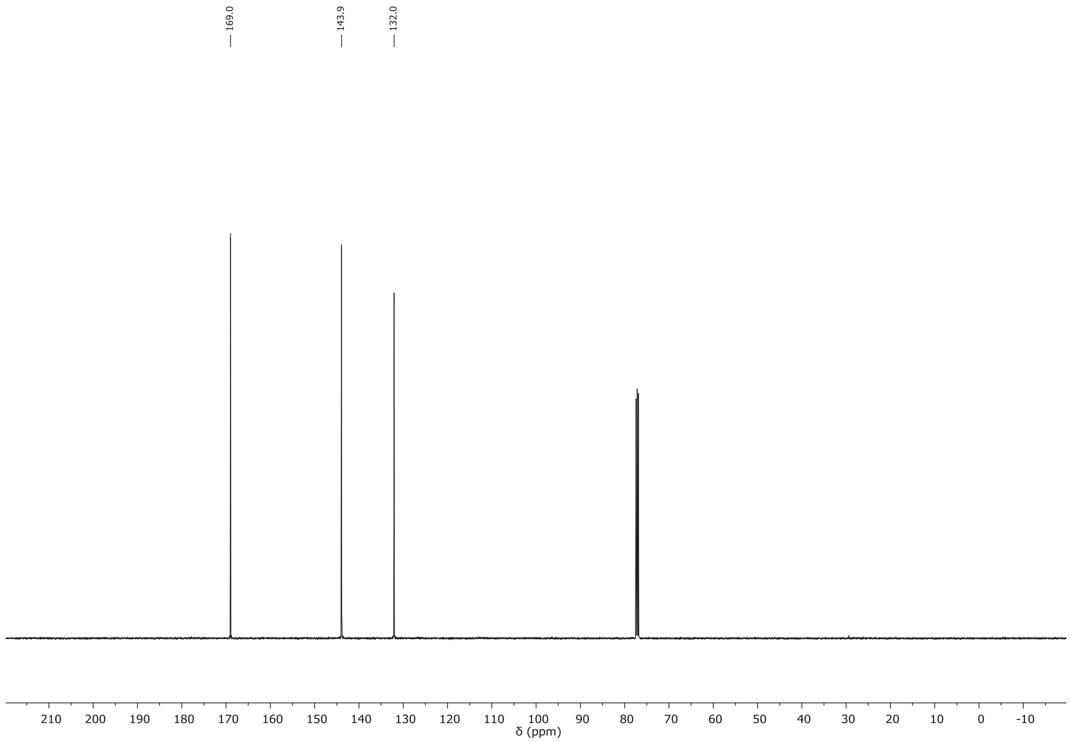

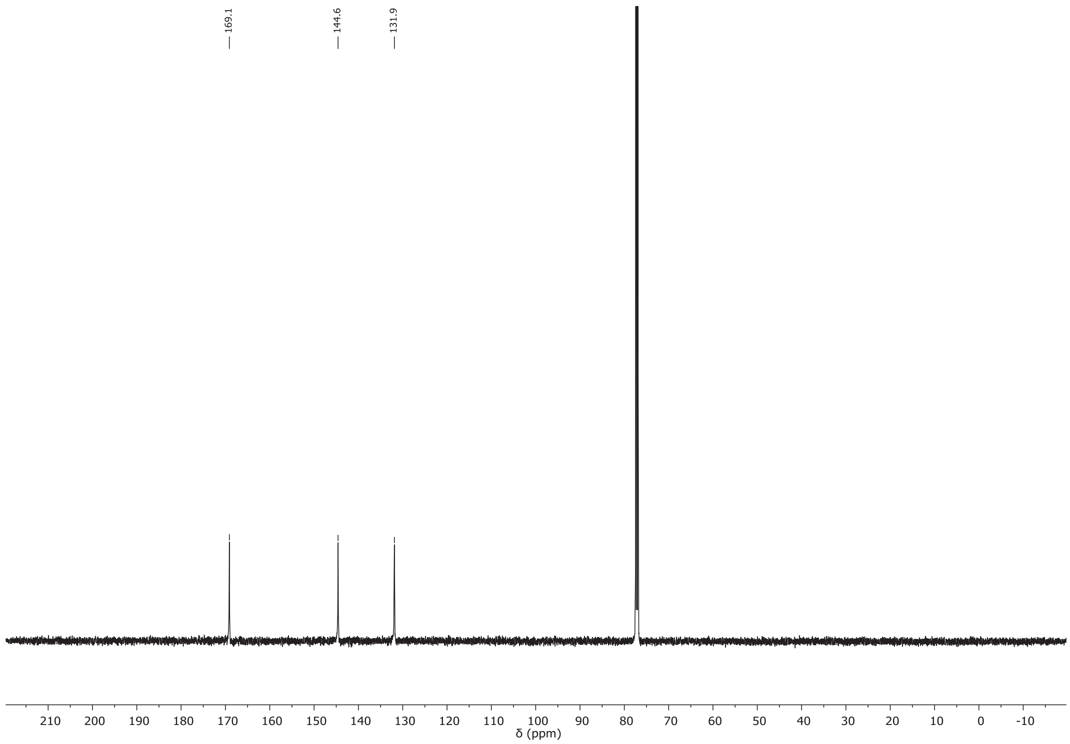

Figure S1. $^{13}C\{^1H\}$ NMR spectra (126 MHz, $CDCl_3$) of the *in-situ* reaction of *o*-chloranil (top) when mixed with one equivalent of $SbBr_3$ (bottom).

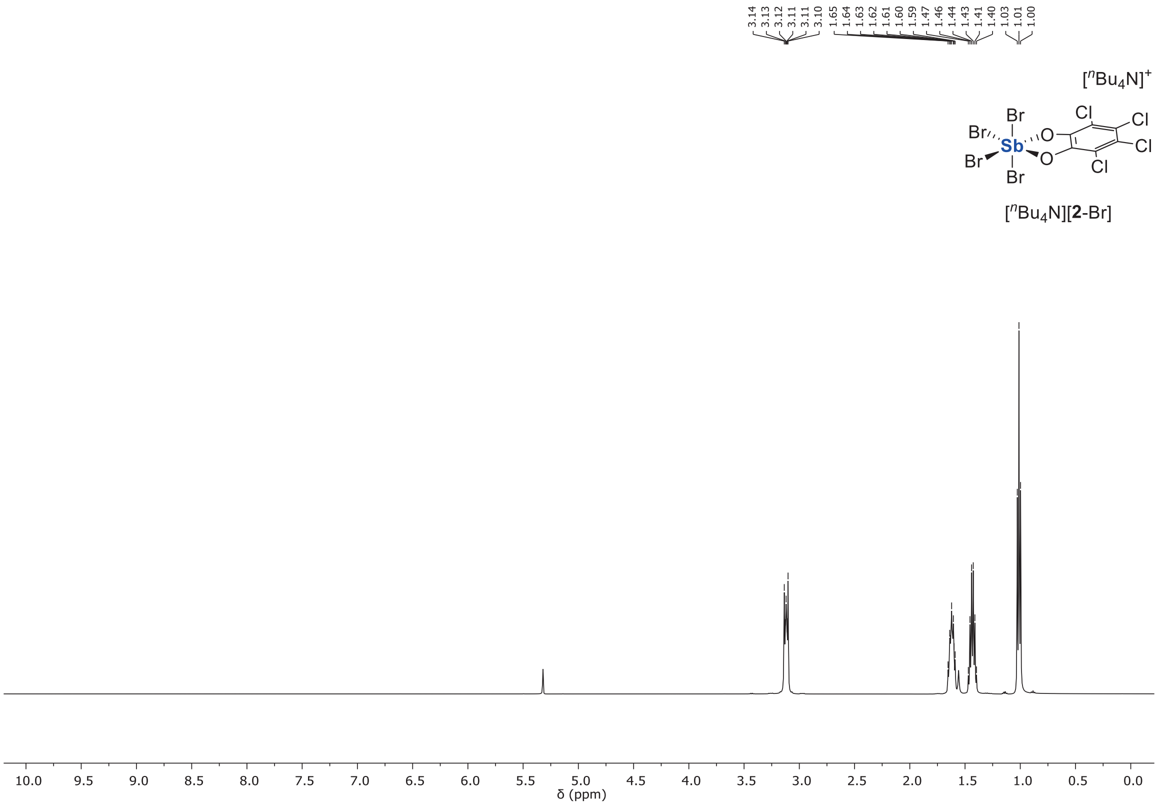

Figure S2. ^{1}H NMR (500 MHz, CD_2Cl_2) spectrum of crude [^{n}Bu$_4$N][**2**-Br].

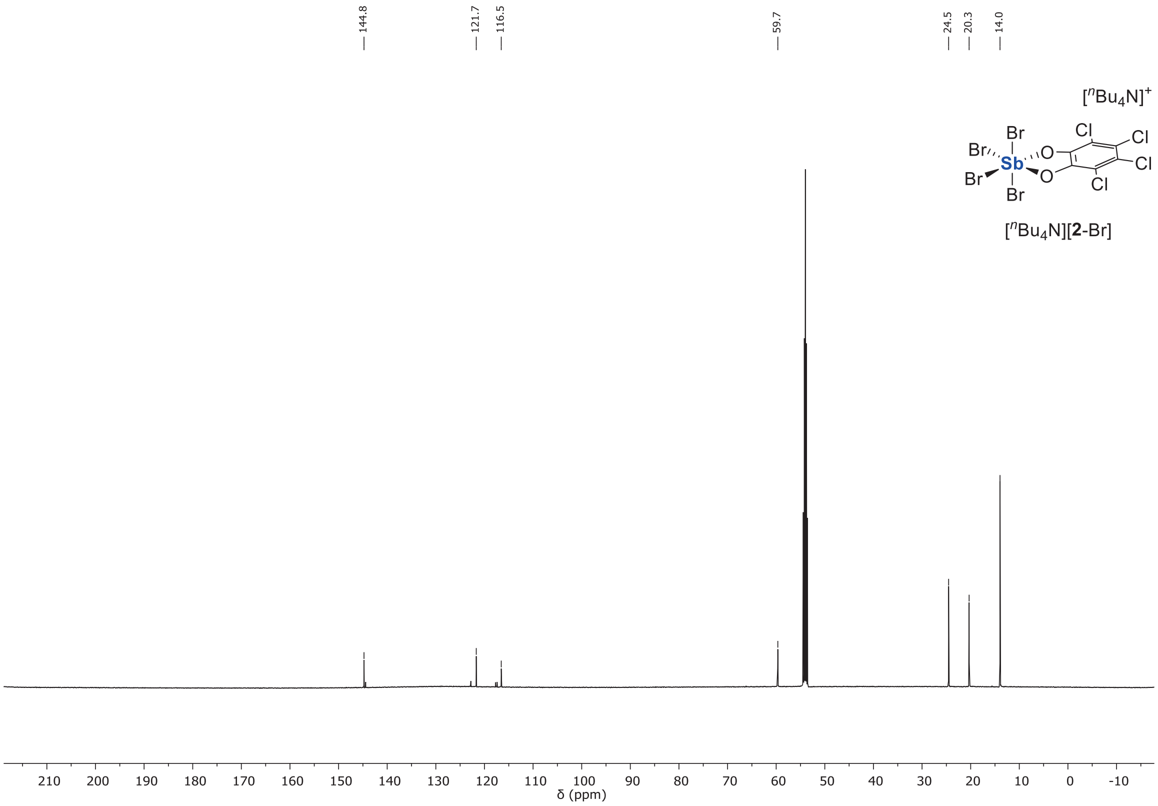

Figure S3. $^{13}C\{^{1}H\}$ NMR (126 MHz, $CDCl_3$) spectrum of crude [$^{n}Bu_4N$][**2**-Br].

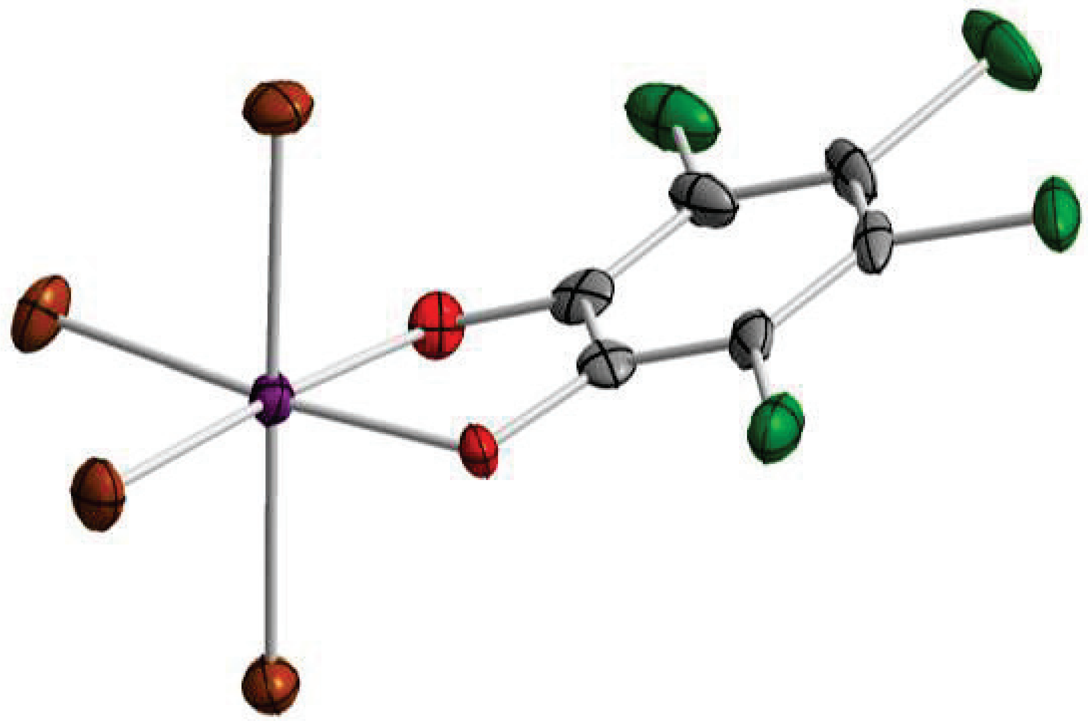

Figure S4. Solid-state structure of [^{n}Bu$_4$N][**2**-Br]. [^{n}Bu$_4$N]$^+$ counterion omitted for clarity. (Gray: C, green: Cl, red: O, purple: Sb, bronze: Br).

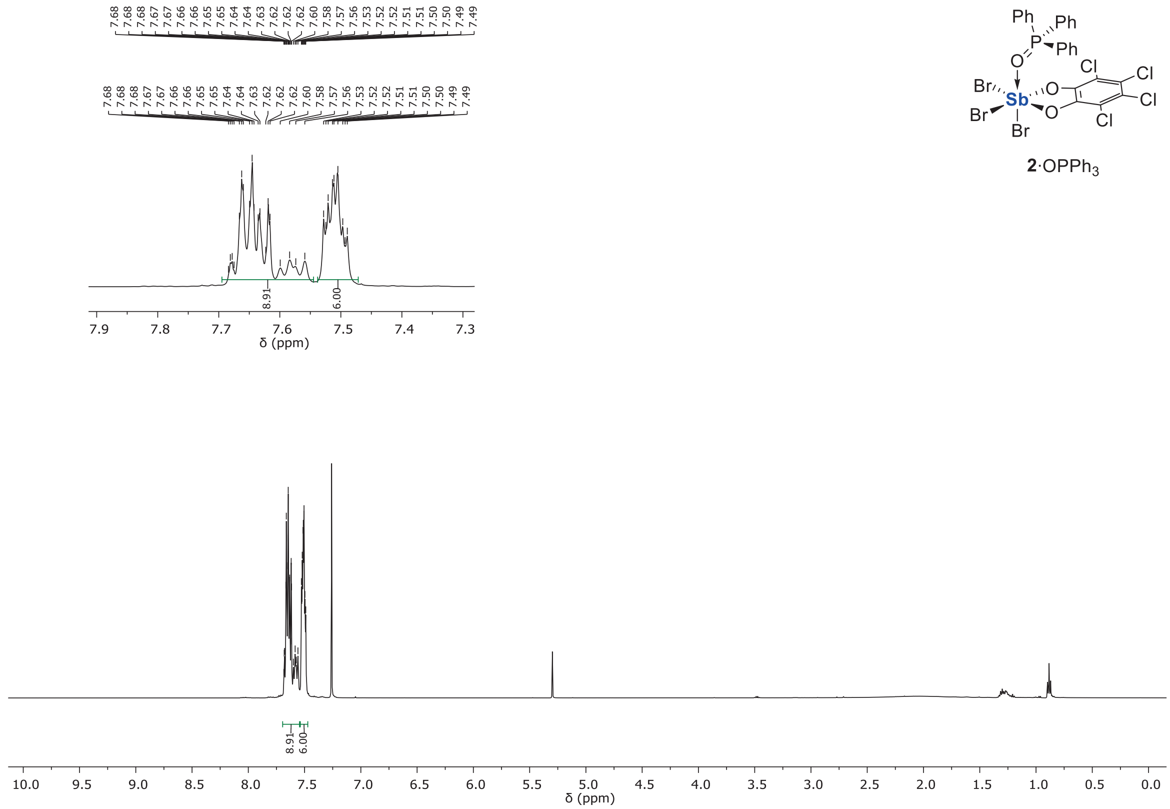

Figure S5. ^{1}H NMR (500 MHz, $CDCl_3$) spectrum of **2**•$OPPh_3$.

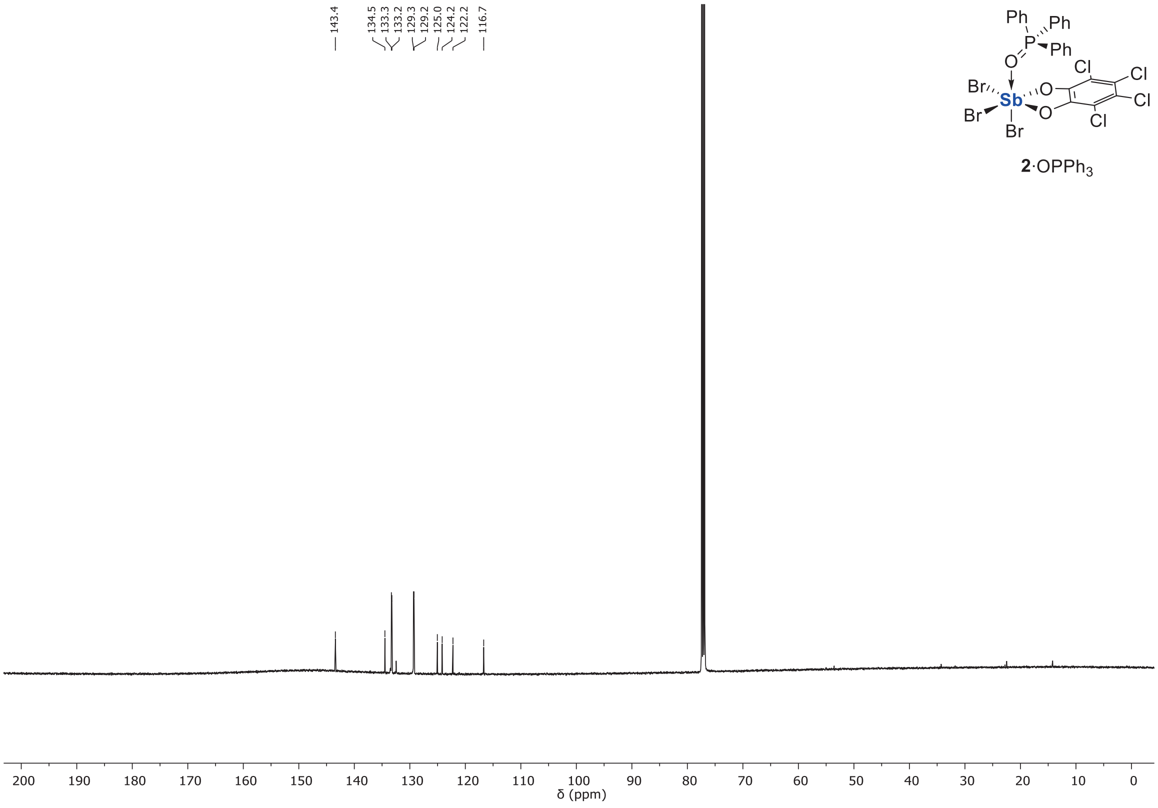

Figure S6. $^{13}C\{^{1}H\}$ NMR (126 MHz, $CDCl_3$) spectrum of **2**•$OPPh_3$. Solvent peak is truncated for clarity.

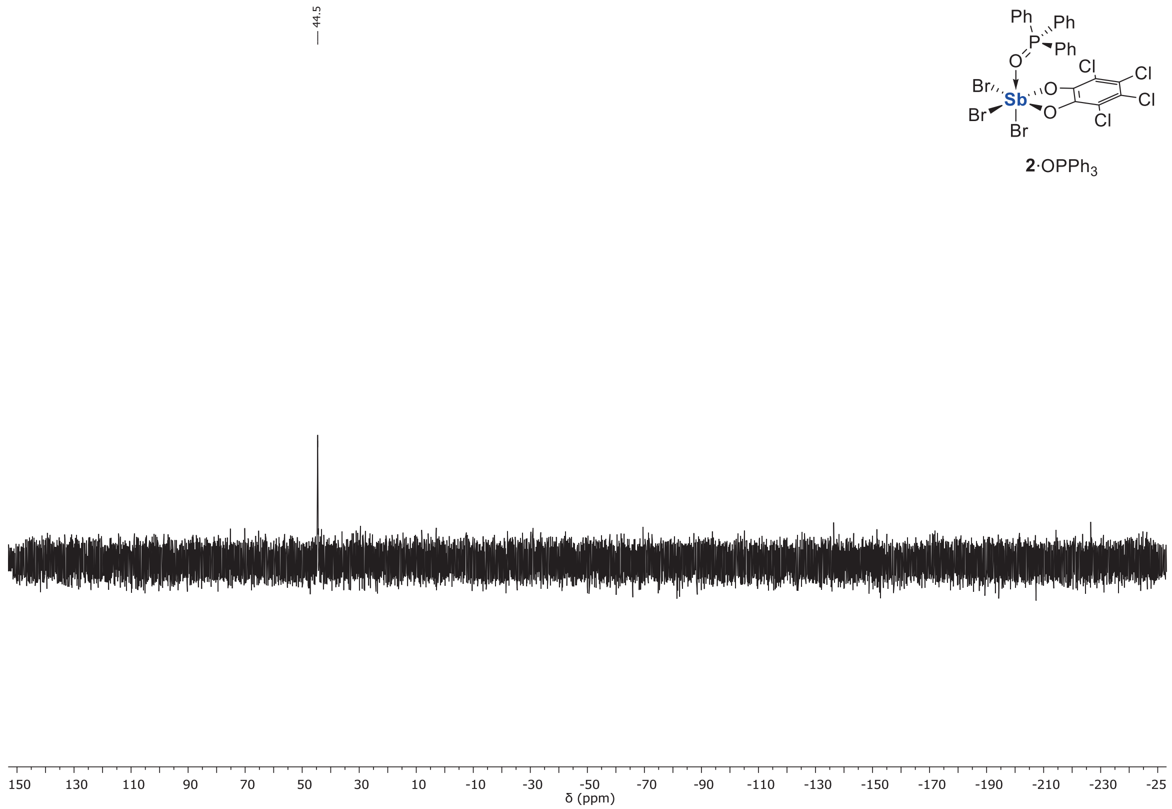

Figure S7. $^{31}P\{^1H\}$ NMR (162 MHz, $CDCl_3$) spectrum of **2**•$OPPh_3$.

2. Computational methods

2.1. Geometry optimizations

All compounds were optimized in Orca 6.0.1[7] using PBEh-3c/def2-mSVP[8] with the default *defgrid2* settings. All geometries were identified as local minima using frequency calculations at the same level of theory.

2.2. *In silico* reaction thermochemistry

Single-point energies for the optimized geometries were performed in Orca 6.0.1 using the DSD-BLYP functional[9] with Grimme's empirical D3 correction using the Becke-Johnson (BJ) damping function[10] with the def2-QZVPP basis set[11] using the RIJCOSX approximation. To obtain enthalpies and Gibbs free energies from these electronic energies, the necessary thermochemical corrections were added from the frequency calculations used to determine that the geometries were minima.

The thermodynamic properties of the *in silico* reactions between $SbCl_3$/$SbBr_3$ and *o*-chloranil were determined from this data.

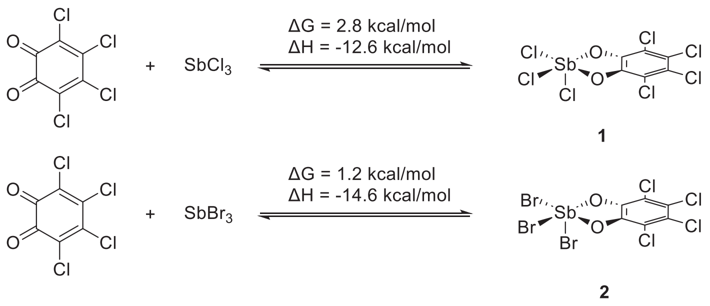

In addition, the thermodynamic properties of the *in silico* reactions between **1**/**2** and dimethyl ether (OMe_2) were determined.

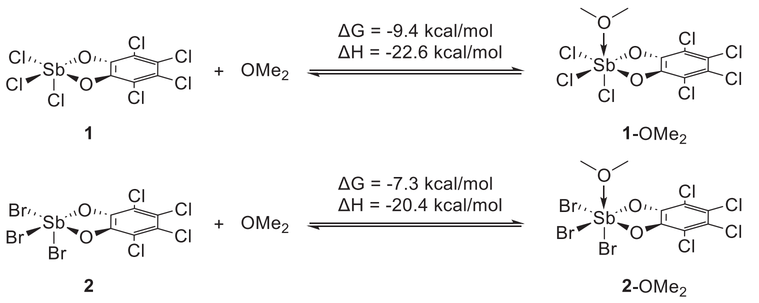

The ΔG of the isodesmic reaction between **1** and **2**•OMe_2 as follows was determined.

$$\mathbf{1} + \mathbf{2}\bullet OMe_2 \rightleftharpoons \mathbf{2} + \mathbf{1}\bullet OMe_2 \quad \Delta G = -2.1\ kJ/mol$$

2.3. Energy summary

	**1**	*o*-chloranil	$SbCl_3$	**2**
E (Hartree)	-3839.041178	-2218.827166	-1620.191771	-10180.06751
E_{corr} (Hartree)	0.07241942	0.06001366	0.00936089	0.07181937
H_{corr} (Hartree)	0.00094421	0.00094421	0.00094421	0.00094421
S_{corr} (Hartree)	0.06407584	0.04940823	0.03933764	0.06710254
H (Hartree)	-3838.967814	-2218.766208	-1620.181465	-10179.99475
G (Hartree)	-3839.03189	-2218.815616	-1620.220803	-10180.06185

	$SbBr_3$	OMe_2	**1**•OMe_2	**2**•OMe_2
E (Hartree)	-7961.215182	-154.922547	-3994.002562	-10335.025296
E_{corr} (Hartree)	0.00890257	0.086434	0.16268349	0.16197069
H_{corr} (Hartree)	0.00094421	0.00094421	0.00094421	0.00094421
S_{corr} (Hartree)	0.0427481	0.03047795	0.0735321	0.07677798
H (Hartree)	-7961.205336	-154.835169	-3993.838934	-10334.862381
G (Hartree)	-7961.248084	-154.865647	-3993.912466	-10334.939159

3. Catalytic screening

3.1. General procedure for the C-O bond activation of diglyme

In a J. Young NMR tube, the appropriate antimony trihalide (0.037 mmol) and *o*-chloranil (9 mg, 0.037 mmol) were dissolved in 0.5 mL of *o*-dichlorobenzene under nitrogen, after which diglyme (106 µL, 0.74 mmol) was added to the solution. The reaction mixture was then heated at 115 °C for 19 h. Upon completion of the reaction, 0.1 mL of $CDCl_3$ was added to the mixture, which was subsequently analyzed by NMR spectroscopy.

3.2. General procedure for the depolymerization of poly(cyclohexene carbonate)

In a J. Young NMR tube, 46 mg of poly(cyclohexene carbonate), the appropriate antimony trihalide (0.016 mmol) and *o*-chloranil (4 mg, 0.016 mmol) were dissolved in 0.5 mL of toluene under nitrogen. The reaction mixture was then heated at 110 °C for 2 h, 4 h, and 10 h. Upon completion of the reaction, 0.1 mL of $CDCl_3$ was added to the mixture, which was subsequently analyzed by NMR spectroscopy.

3.3. Characterization of the products

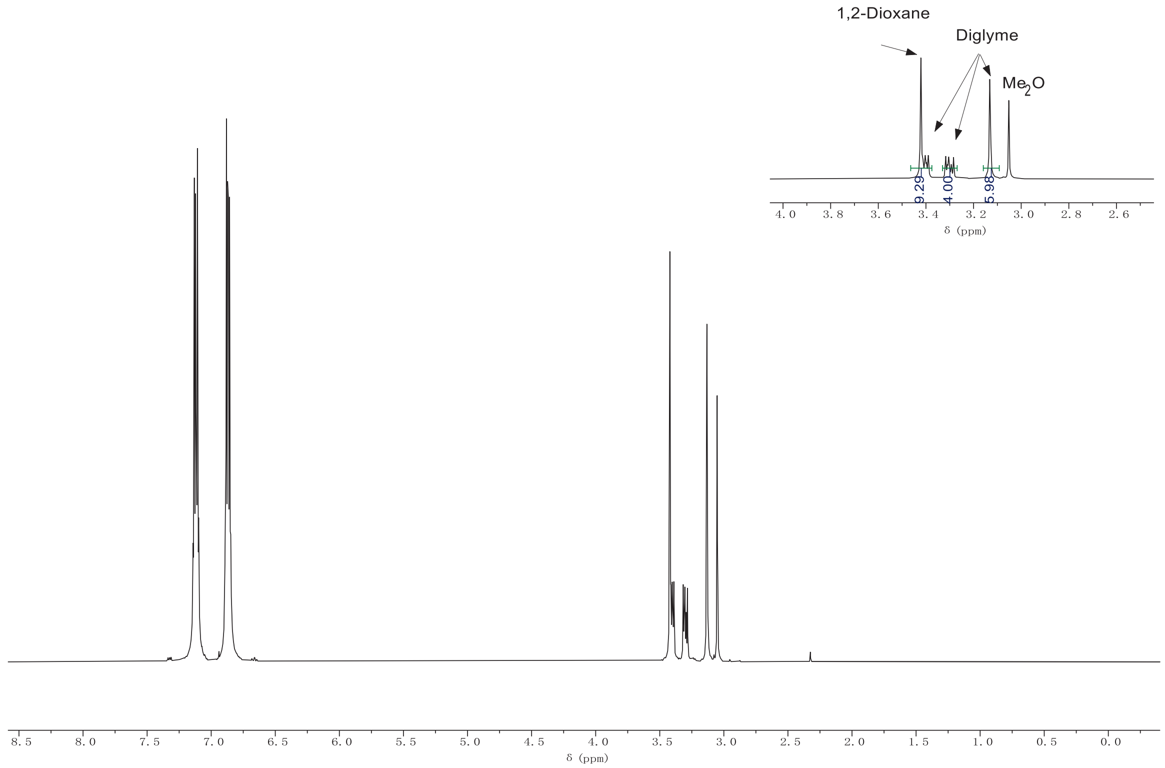

Figure S8. ^{1}H NMR spectrum of the crude reaction mixture obtained by reaction of diglyme with the $SbBr_3$/*o*-chloranil system.

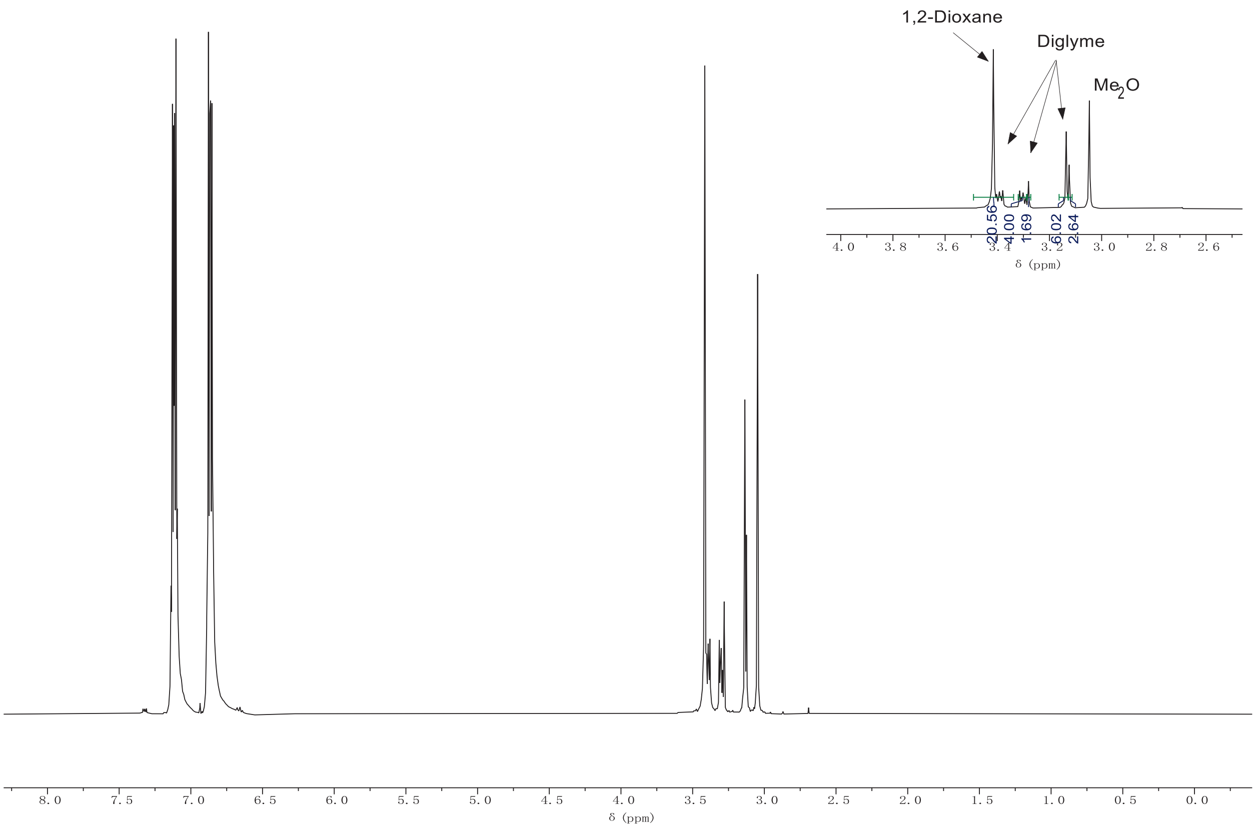

Figure S9. ^{1}H NMR spectrum of the crude reaction mixture obtained by reaction of diglyme with the $SbCl_3$/*o*-chloranil system.

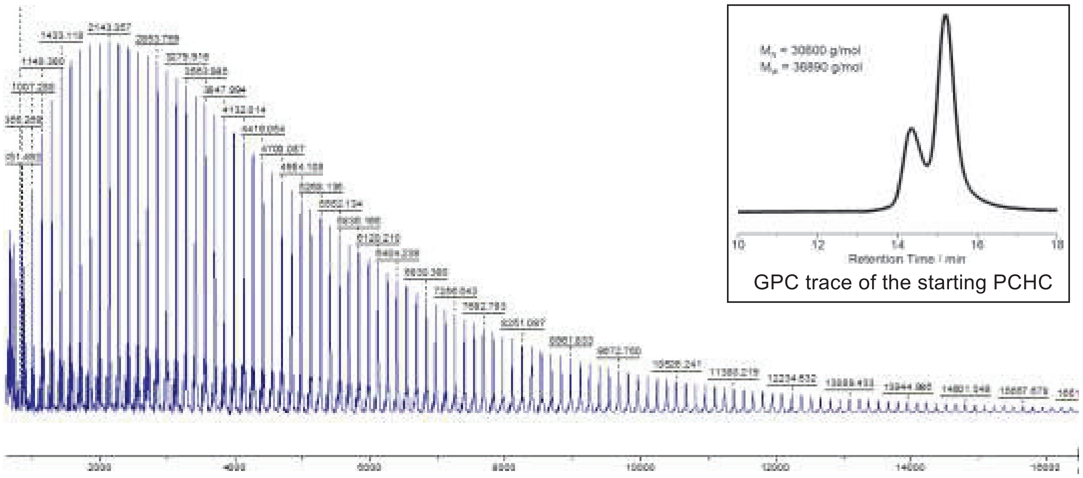

Figure S10. MALDI-TOF mass spectrum of the polymer residue obtained after the depolymerization reaction of poly(cyclohexene carbonate) promoted by the $SbBr_3$/*o*-chloranil system.

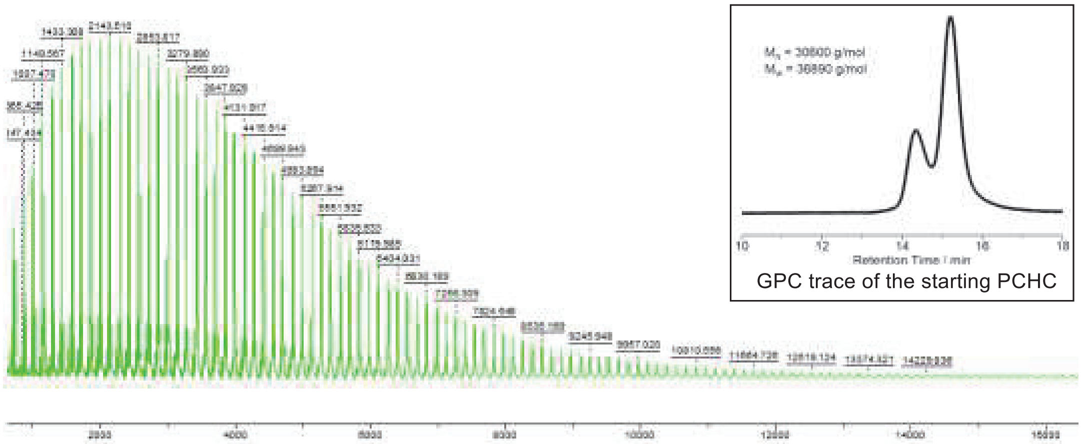

Figure S11. MALDI-TOF mass spectrum of the polymer residue obtained after the depolymerization reaction of poly(cyclohexene carbonate) promoted by the $SbCl_3$/*o*-chloranil system.

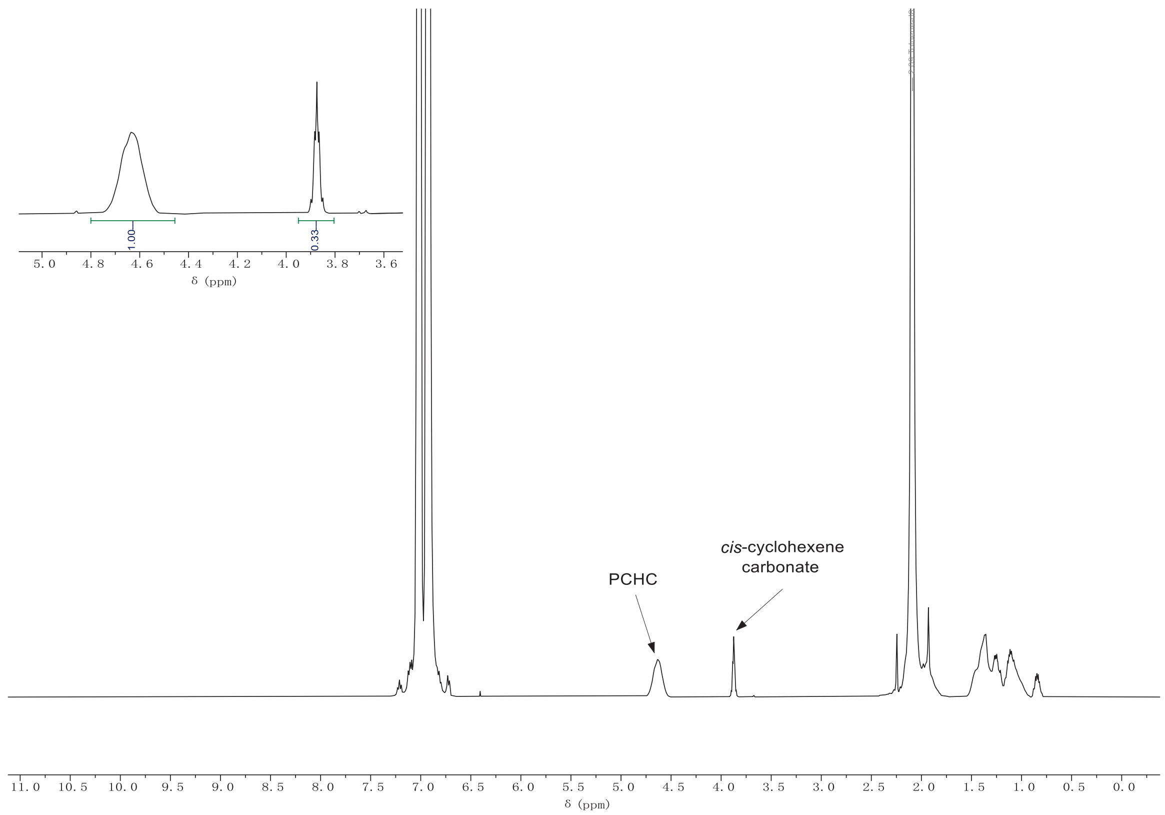

Figure S12. ^{1}H NMR spectrum of the crude reaction mixture recorded during depolymerization reaction of poly(cyclohexene carbonate) promoted by the $SbBr_3$/*o*-chloranil system after 2 h.

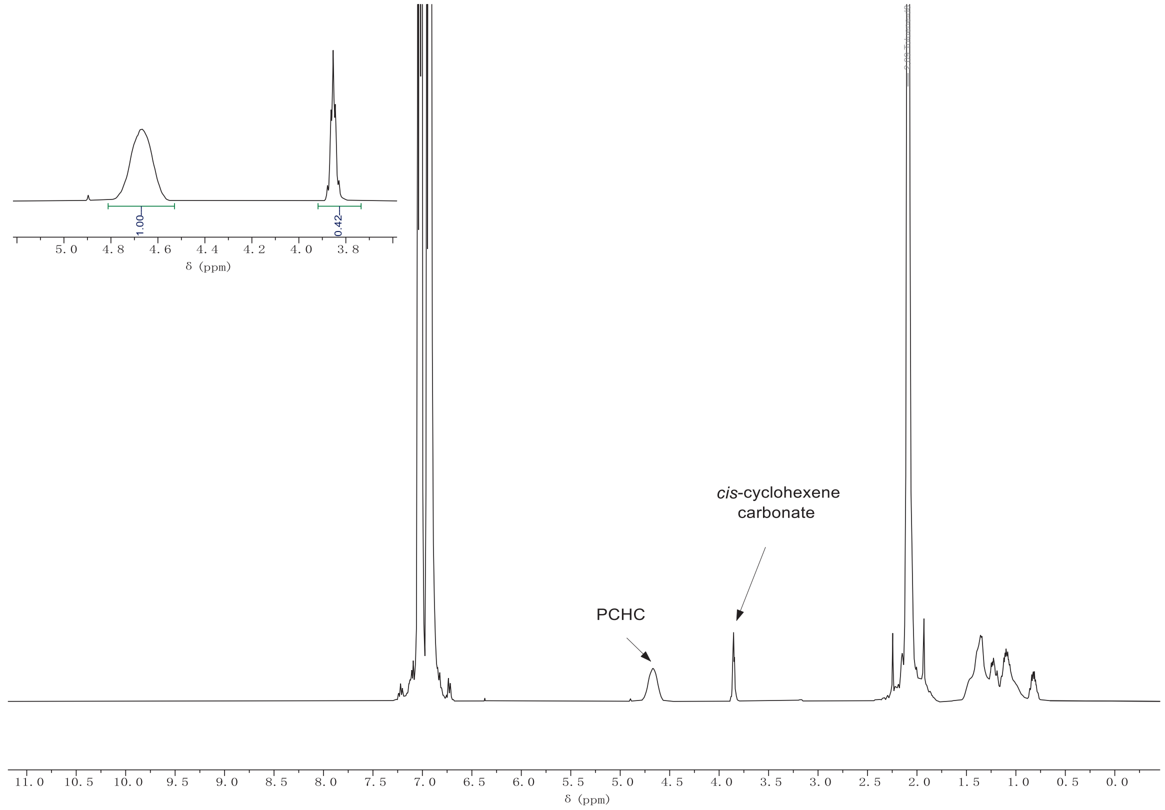

Figure S13. ^{1}H NMR spectrum of the crude reaction mixture recorded during depolymerization reaction of poly(cyclohexene carbonate) promoted by the $SbCl_3$/*o*-chloranil system after 2 h.

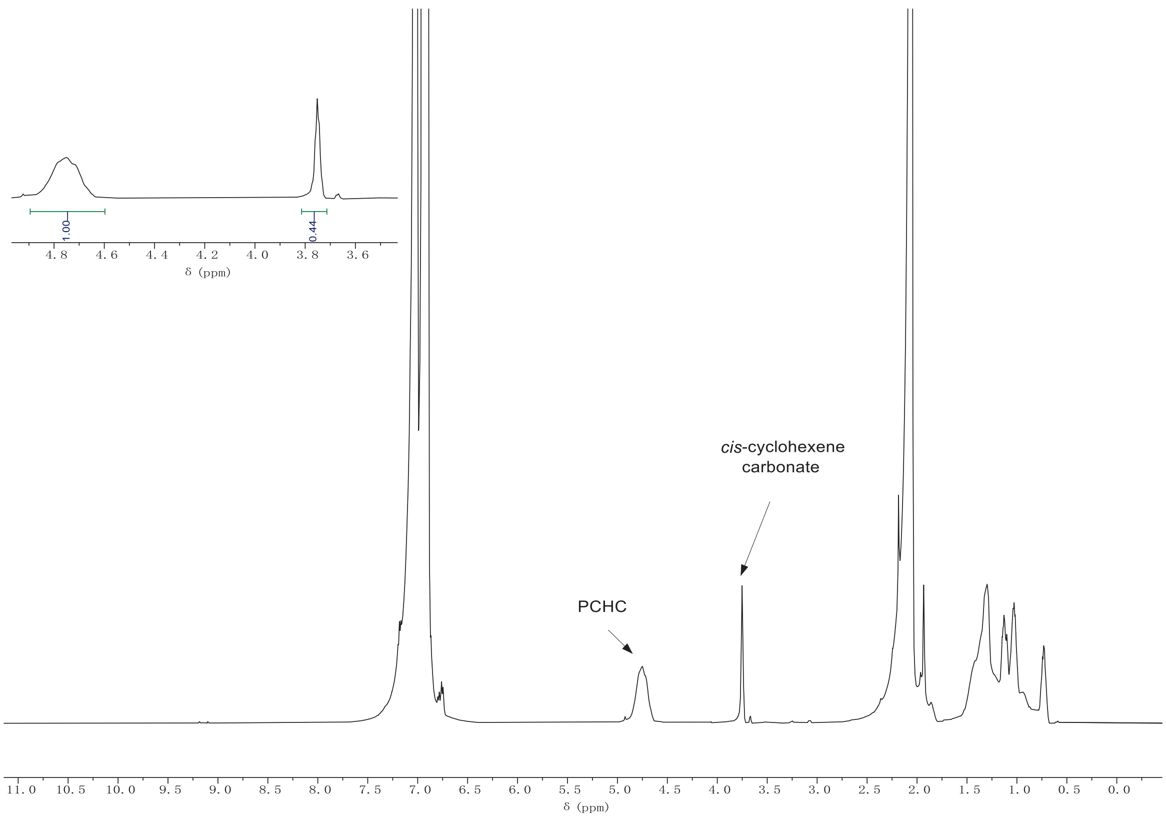

Figure S14. ^{1}H NMR spectrum of the crude reaction mixture recorded during depolymerization reaction of poly(cyclohexene carbonate) promoted by the $SbBr_3$/*o*-chloranil system after 4 h.

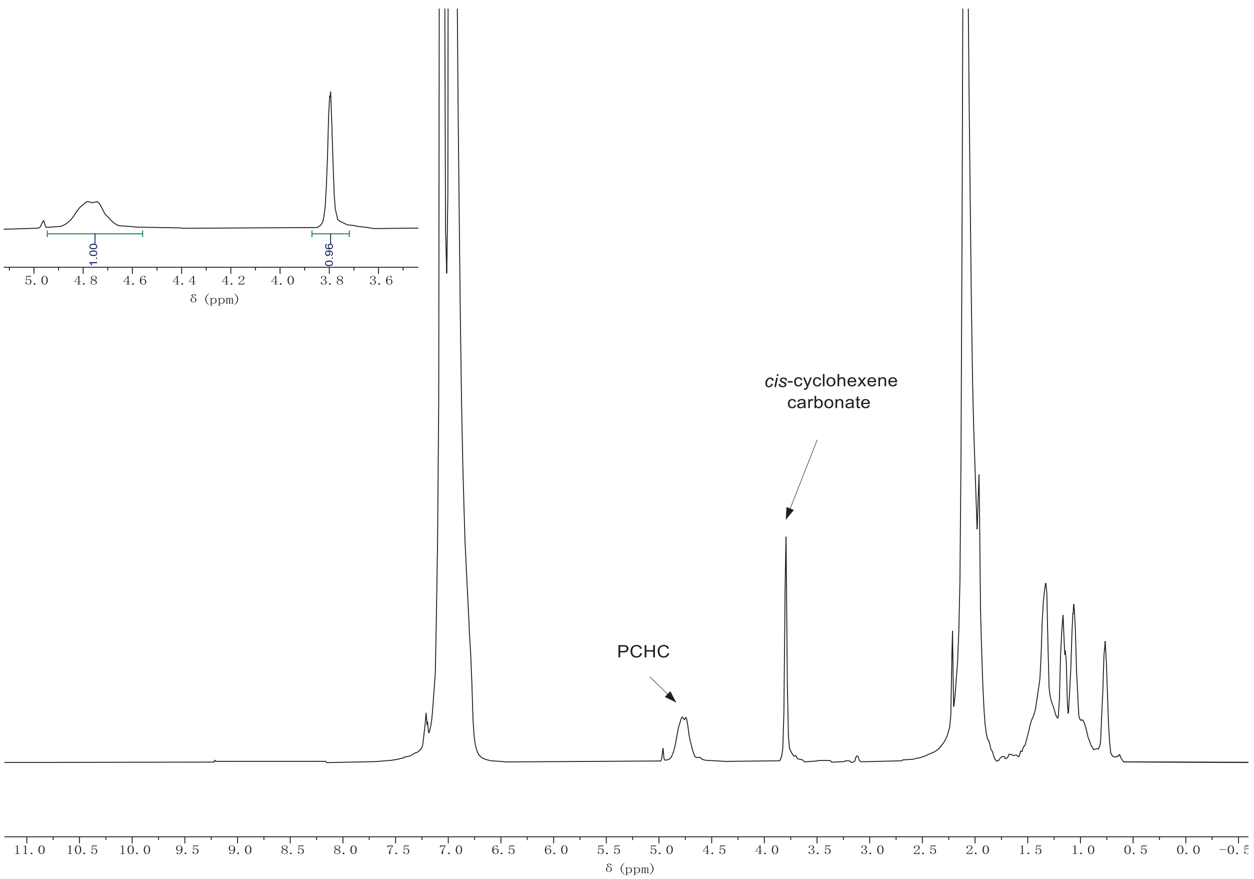

Figure S15. ^{1}H NMR spectrum of the crude reaction mixture recorded during depolymerization reaction of poly(cyclohexene carbonate) promoted by the $SbCl_3$/*o*-chloranil system after 4 h.

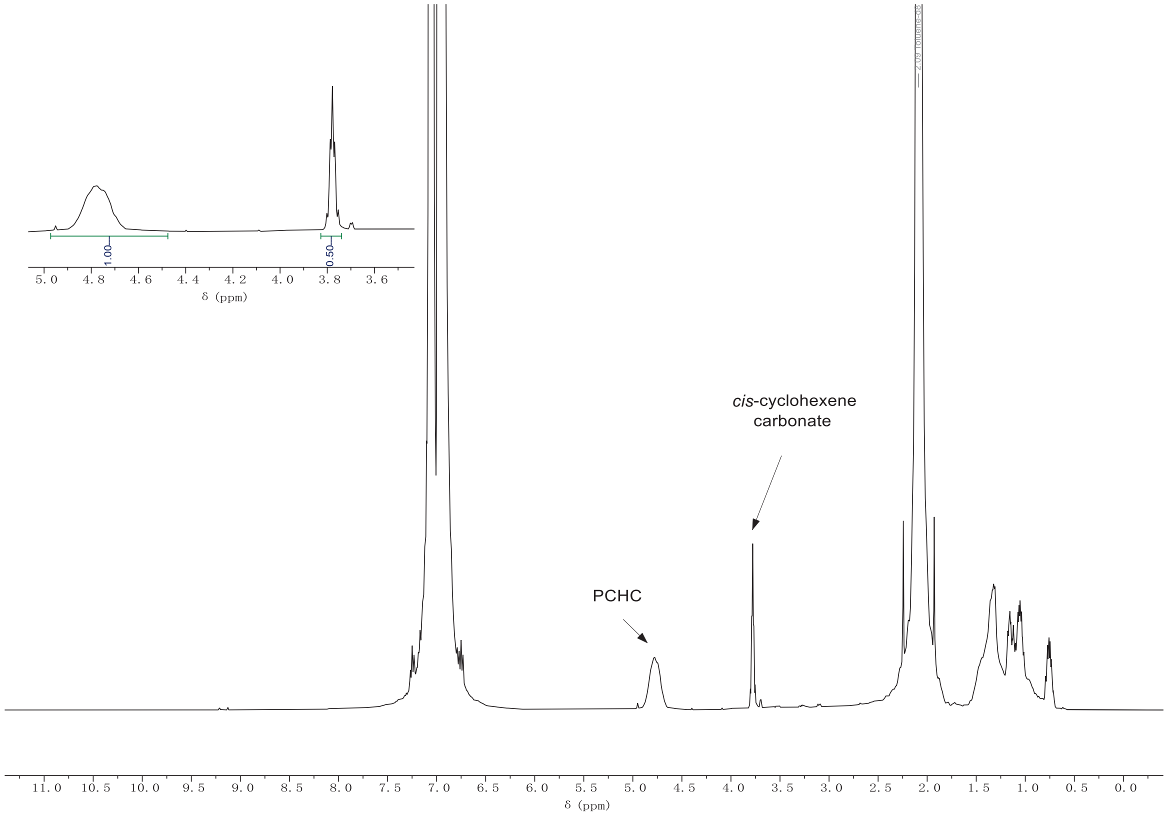

Figure S16. ^{1}H NMR spectrum of the crude reaction mixture recorded during depolymerization reaction of poly(cyclohexene carbonate) promoted by the $SbBr_3$/*o*-chloranil system after 10 h.

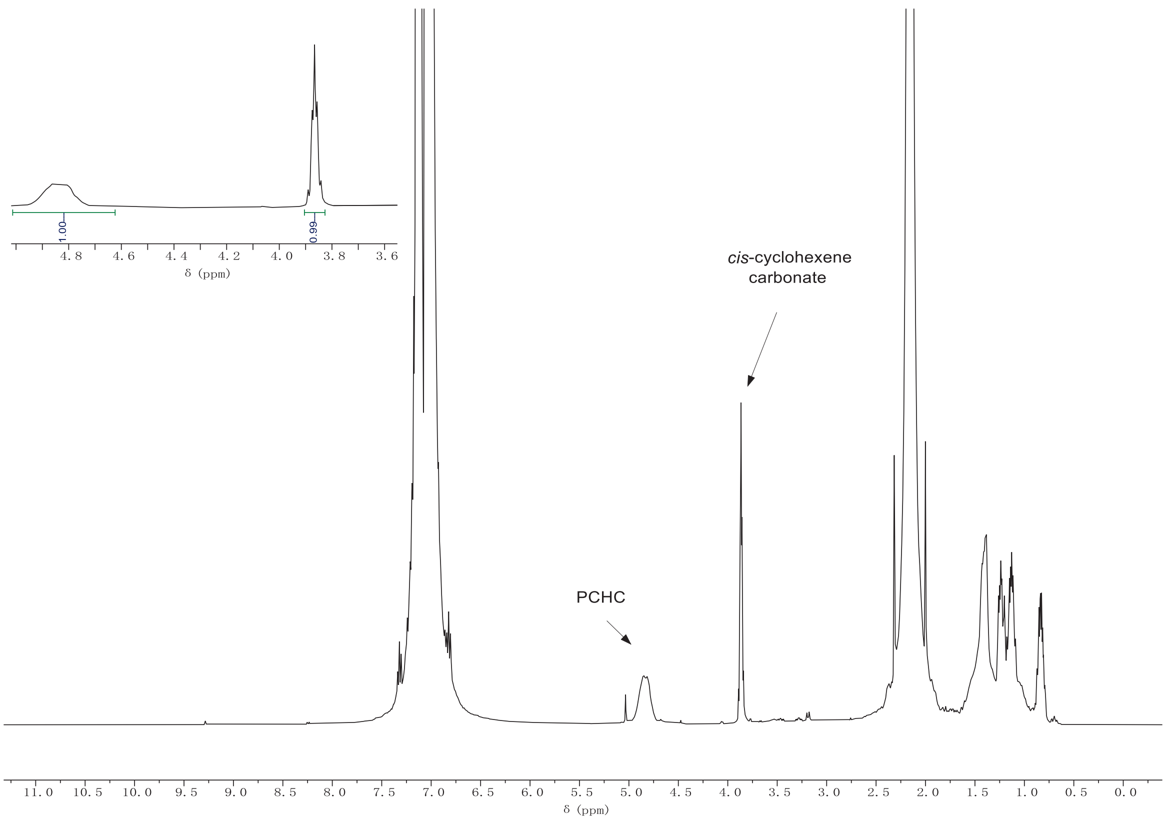

Figure S17. ^{1}H NMR spectrum of the crude reaction mixture recorded during depolymerization reaction of poly(cyclohexene carbonate) promoted by the $SbCl_3$/*o*-chloranil system after 10 h.

References

1. Jiang, C.; Lee, E.; Schaefer, J.; Holtcamp, M. W.; Lin, T.-P.; Gabbaï, F. P. Pnictogen-Bonding Catalysis: Copolymerization of CO_2 and Epoxides on Antimony(V) Platforms. *ACS Catal.* **2025**, *15*, 17882-17892.
2. Bruker, 2019, *APEX3*, Version 2019.0-1, Bruker AXS Inc., Madison, Wisconsin, USA.
3. Sheldrick, G. M. *SADABS,* Version 2007/4, Bruker Analytical X-ray Systems, Inc.: Madison, Wisconsin, USA, 2007.
4. Sheldrick, G. M. SHELXT - Integrated space-group and crystal-structure determination. *Acta Crystallogr., Sect. A: Found Adv.* **2015**, *71*, 3-8.
5. Sheldrick, G. M. *SHELXL-2014: Program for Crystal Structure Refinement*, University of Göttingen, Germany, 2014.
6. Dolomanov, O. V.; Bourhis, L. J.; Gildea, R. J.; Howard, J. A. K.; Puschmann, H. OLEX2: a complete structure solution, refinement and analysis program. *J. Appl. Crystallogr.* **2009**, *42*, 339-341.
7. Neese, F. Software Update: The ORCA Program System—Version 6.0. *WIREs Comput. Mol. Sci.* **2025**, *15*, e70019.
8. Grimme, S.; Brandenburg, J. G.; Bannwarth, C.; Hansen, A. Consistent structures and interactions by density functional theory with small atomic orbital basis sets. *J. Chem. Phys.* **2015**, *143*, 054107.
9. Kozuch, S.; Gruzman, D.; Martin, J. M. L. DSD-BLYP: A General Purpose Double Hybrid Density Functional Including Spin Component Scaling and Dispersion Correction. *J. Phys. Chem. C* **2010**, *114*, 20801-20808.
10. Grimme, S.; Ehrlich, S.; Goerigk, L. Effect of the damping function in dispersion corrected density functional theory. *J. Comput. Chem.* **2011**, *32*, 1456-1465.
11. Weigend, F.; Ahlrichs, R. Balanced basis sets of split valence, triple zeta valence and quadruple zeta valence quality for H to Rn: Design and assessment of accuracy. *Phys. Chem. Chem. Phys.* **2005**, *7*, 3297-3305.
